# Gait analysis with the Kinect v2: normative study with healthy individuals and comprehensive study of its sensitivity, validity, and reliability in individuals with stroke

**DOI:** 10.1186/s12984-019-0568-y

**Published:** 2019-07-26

**Authors:** Jorge Latorre, Carolina Colomer, Mariano Alcañiz, Roberto Llorens

**Affiliations:** 1 Neurorehabilitation and Brain Research Group, Instituto de Investigación e Innovación en Bioingeniería, Universitat Politècnica de València, Camino de Vera s/n, 46022 Valencia, Spain; 2 NEURORHB, Servicio de Neurorrehabilitación de Hospitales Vithas, Río Tajo 1, 46011 Valencia, Spain

**Keywords:** Gait, Stroke, Biomedical technology assessment, Reliability and validity, Fall risk, Kinect v2

## Abstract

**Background:**

Gait is usually assessed by clinical tests, which may have poor accuracy and be biased, or instrumented systems, which potentially solve these limitations at the cost of being time-consuming and expensive. The different versions of the Microsoft Kinect have enabled human motion tracking without using wearable sensors at a low-cost and with acceptable reliability. This study aims: First, to determine the sensitivity of an open-access Kinect v2-based gait analysis system to motor disability and aging; Second, to determine its concurrent validity with standardized clinical tests in individuals with stroke; Third, to quantify its inter and intra-rater reliability, standard error of measurement, minimal detectable change; And, finally, to investigate its ability to identify fall risk after stroke.

**Methods:**

The most widely used spatiotemporal and kinematic gait parameters of 82 individuals post-stroke and 355 healthy subjects were estimated with the Kinect v2-based system. In addition, participants with stroke were assessed with the Dynamic Gait Index, the 1-min Walking Test, and the 10-m Walking Test.

**Results:**

The system successfully characterized the performance of both groups. Significant concurrent validity with correlations of variable strength was detected between all clinical tests and gait measures. Excellent inter and intra-rater reliability was evidenced for almost all measures. Minimal detectable change was variable, with poorer results for kinematic parameters. Almost all gait parameters proved to identify fall risk.

**Conclusions:**

Results suggest that although its limited sensitivity to kinematic parameters, the Kinect v2-based gait analysis could be used as a low-cost alternative to laboratory-grade systems to complement gait assessment in clinical settings.

## Background

The physiological basis of cerebrovascular accidents make gait deficits a common sequelae after stroke [1]. More than 60% of stroke survivors are unable to walk independently after the injury [2] and, even after rehabilitation, more than half of the cases still present gait-related deficits [3]. Most prevailing deficits after stroke include reduced speed [4] and increased gait inter-limb asymmetry [5]. These gait impairments can be aggravated in the elderly, due to the natural musculoskeletal and cognitive decline with age [6, 7], where the incidence of stroke is higher [8]. Importance of these deficits relies on their great impact on independence [9], quality of life [10], and fall risk [11]. Consequently, their adequate assessment is necessary for a proper diagnosis and to plan, if required, customized interventions to each individual’s condition and evaluate the effectiveness of these interventions.

Assessment of gait is commonly performed in the clinical setting using standardized scales and tests that evaluate different aspects of human locomotion and, in some cases, compare the results of the person being tested with those obtained by a matched healthy sample [12]. Although these tools are easy to administer and, in general, not time-consuming, they can present lack of specificity and, more importantly, may have poor accuracy and be biased by subjective evaluations [13]. Over the years, different technological solutions have been proposed to overcome these limitations. Accurate estimation of spatiotemporal parameters has been enabled by instrumented walkways [14] and force plates [15], generally, from ground reaction forces during walking. Estimation of kinematic parameters, however, require the position of several joints to be tracked during the test, which has been indirectly facilitated by different technological solutions that estimate the position of some sensors that are attached to specific body parts [16–18]. Among them, optical motion tracking has become the most common alternative for accurate investigation of kinematic gait parameters [19]. Although instrumented systems allow for accurate spatiotemporal and kinematic analysis, their high cost and large size have restricted their use to research laboratories and large clinical centers with high economic resources [20].

In the last years, the Microsoft Kinect (Microsoft, Redmond, WA), a portable off-the-shelf infrared camera originally intended for entertainment, has enabled human motion tracking without using wearable sensors at a very low-cost. Reliability studies have shown comparable performance of the Kinect to laboratory-grade gait analysis systems, for both the first [21, 22] and the second version of the device [23], known as the Kinect v2, which features improved depth accuracy and number of joints tracked [24]. Characteristics of the Kinect v2 have motivated their use for assessing spatiotemporal [25–27] and kinematic parameters of gait [26, 28] with promising results in healthy individuals, even on treadmills [28, 29]. Its reliability in stroke population, however, remains almost unexplored. Little evidence suggests that data retrieved from the Kinect v2 can be used to differentiate healthy subjects from individuals with stroke [30] and to complement clinical assessment [31]. Despite of the existing data supporting the reliability of the Kinect v2 to assess spatiotemporal and kinematic gait parameters, the unavailability of the software, the limited investigation in individuals with stroke, and the unknown psychometric properties of Kinect-based tests in this population could compromise the clinical relevance of these results.

The objective of this study was fourfold. First, to compare a cohort of individuals with stroke with respect to a group of healthy controls to determine the sensitivity of an open-access Kinect v2-based gait analysis system to motor disability and aging. Second, to determine the concurrent validity of the system with standardized clinical tests in individuals with stroke. Third, to quantify its reliability as defined by the inter and intra-rater reliability, the standard error of measurement, and the minimal detectable change. And, finally, to investigate the ability of the system to identify risk of falls after stroke.

## Methods

### Participants

Individuals with stroke were recruited from the outpatient service of a long-term neurorehabilitation unit. Inclusion criteria in this group were: 1) age ≥ 10; 2) ability to walk 10 m indoors with or without assistance; and 3) ability to understand instructions (Mini-Mental State Examination [32]> 23). Exclusion criteria were: 1) individuals with severe aphasia (Mississippi Aphasia Screening Test [33]< 45); 2) individuals with permanent fixed contracture of joints in the legs; 3) individuals with arthritic or orthopedic conditions affecting the lower limbs; and 4) individuals with severe hemispatial neglect. A sample size of 82 post-stroke participants was calculated for an effect size of 0.3, a power of 0.80, an alpha of 0.05, and two tails.

Healthy subjects older than 10 years old with no known musculoskeletal or vestibular disease and/or prosthetic surgery were potential candidates to participate in the study. A minimum sample size of 328 healthy participants, four times the number of participants post-stroke was required. A minimum of 40 healthy participants per decade was also required to avoid a heterogeneous distribution.

Ethical approval for the study was granted by the Institutional Review Board of Vithas Hospital Valencia al Mar. All eligible candidates who agreed to take part in the study provided written informed consent.

### Instrumentation

A Kinect v2 was used to retrieve the 3D pose of the participants and to provide a RGB video of their performance at 30 Hz during the assessment [34] using the Kinect for Windows Software Development Kit 2.0. An MSI GT70-066ES (Micro-Star International Co., Ltd., Zhonghe, New Taipei, Taiwan) which included an 8-core Intel® Core™ i7-3610QM @3.30 GHz, 8 GB of RAM, and NVIDIA® GeForce® GTX670M (Nvidia Corporation, Santa Clara, CA, USA) run a custom-made gait analysis software.

A dedicated application was designed to register the 3D position and orientation of the 25 human joints provided by the Kinect v2 while they approach to the device from the maximum (5 m) to the minimum distance covered (0.5 m), it is, for a total of 4.5 m [35]. After each trial, the application estimates and stores the most widely used spatiotemporal and kinematic gait parameters. Parameters that involved ankle kinematics were discarded, as the Kinect v2 has been reported to have poor reliability in ankle detection [36] and step asymmetry was calculated as the difference between right and left step length. All other kinematic parameters, and the speed, stride, and step measures were computed from the speed of the ankles. Shorter events were estimated from the distance between the sacrum and the ankles and toes. These methods have been shown to provide the best accuracy from data retrieved by the Kinect v2 [31].

### Procedure

Participants were assessed in dedicated areas, clear and free of distractors. Use of close-fitting, pale, and non-reflective clothes was indicated to limit the tracking errors. Participants were initially located at a distance of 6 m from the Kinect v2 in its longitudinal axis. Initial position was marked on the floor using a tape strap. An experimenter asked participants to walk towards the camera until reaching the device at a comfortable speed with a self-initiated movement. Prior to the experiment, all the participants were allowed to practice until they achieved a valid repetition. The position and orientation of the participants’ joints were registered and the gait parameters were computed and provided after each repetition. The experimenter checked the performance of the participants and discarded those repetitions that were affected by tracking errors or unusual performance, such as sudden stops during the repetition. A minimum of three valid repetitions were required to ensure repeatability.

In addition, the gait characteristics of participants with stroke was assessed with the Dynamic Gait Index (DGI) [37, 38], the 1-min Walking Test (1mWT) [39], and the 10-m Walking Test (10MWT) [40]. In addition, their balance was assessed with the Berg Balance Scale (BBS) [41] to determine their risk of falling. All assessments took place within 24 h.

Finally, half the participants post-stroke were randomly considered to estimate the inter and intra-rater reliability. The gait parameters of 21 individuals with were assessed with the Kinect v2-based gait analysis by two different experimenters to determine the inter-rater reliability, and other different 21 subjects were assessed twice by the same experimenter to determine the intra-rater reliability. These tests were also performed within 24 h.

### Data analysis

For each age decade, gait parameters were defined as the average performance of all the subjects in each group. Performance of each subject was computed as his or her average performance in all the existing repetitions. Kinematic parameters were characterized by the range of motion of the joint involved in each parameter. Different statistical analysis were performed to investigate each objective.

First, paired t-tests were used to describe differences in the gait parameters between populations.

Second, Pearson correlation coefficients were calculated to determine the age effect on all measures and the concurrent validity of the Kinect v2-based gait analysis system with clinical tests. Correlations illustrating concurrent validity reflected the extent to which gait measures and clinical tests were related, and consequently, depicted similar motor components.

Third, a two-way random effects model intra-class correlation coefficient with a single rate/measurement (ICC [1, 2]) was used to summarize the strength of the reliability. Values of 0.8 or higher were accepted as indicating very strong reliability. Values in the range of 0.6 to 0.8 and 0.4 to 0.6 were considered indicators of strong and moderate reliability, respectively. Values in the range of 0.2 to 0.4 and below 0.2 were analogously considered indicators of weak and very weak reliability, respectively [42]. The standard error of measurement and the minimal detectable change were also obtained. Minimal detectable change scores higher than 30% were considered poor, from 10 to 30% were considered acceptable, and those lower than 10% were considered excellent [43].

Finally, to determine the ability of the system to identify fall risk in people with stroke, their risk of falling was categorized according to their scores in the BBS. Participants with stroke were classified as fallers (scores below 49) and non-fallers (scores greater or equal to 49) [44], accordingly. Paired t-tests were performed to investigate differences between both groups of participants and a receiver operating characteristic (ROC) area was estimated to determine the ability of each variable to discriminate between fallers and non-fallers. Values of the ROC from 0.9 to 1 were considered excellent, from 0.8 to 0.9 were considered good, from 0.7 to 0.8 were considered fair, from 0.6 to 0.7 were considered poor, and from 0.5 to 0.6 were considered fail [45, 46].

All statistical analyses were performed using IBM SPSS Statistics version 22 (IBM, New York, NY). Two-sided *p*-values below 0.05 were considered statistically significant.

## Results

### Participants

Eighty-two individuals with stroke (55 men and 27 women) participated in the study (Table 1). Post-stroke participants had a mean age of 48.3 ± 16.14 years old and presented either ischemic (*n* = 41) or hemorrhagic stroke (*n* = 41), with a mean time since injury of 748.55 ± 785.12 days.

**Table 1:** Distribution of participants by age and gender

	Healthy subjects		Individuals with stroke					
	Women	Men	Women	Men	Berg Balance Scale	Dynamic gait index	1-min walking test	10-m walking test
10–19	23	24	2	3	53.25 (4.19)	23.60 (0.89)	82.20 (12.97)	7.06 (0.64)
20–29	26	37	4	2	53.17 (4.67)	21.33 (5.13)	64.00 (38.29)	8.48 (3.70)
30–39	22	33	5	6	54.88 (1.36)	22.40 (2.88)	70.90 (16.44)	8.61 (1.34)
40–49	29	14	6	10	51.00 (5.42)	20.81 (3.82)	65.88 (24.44)	10.55 (6.45)
50–59	26	18	6	18	50.61 (7.21)	20.87 (4.24)	58.61 (16.51)	11.43 (4.73)
60–69	28	19	2	11	48.38 (9.45)	18.38 (7.94)	50.31 (23.84)	18.41 (19.99)
≥ 70	32	24	2	5	43.57 (9.50)	17.00 (5.35)	42.86 (12.05)	12.87 (4.48)
Total	186	169	27	55	50.44 (7.29)	20.51 (5.02)	60.75 (22.69)	11.79 (9.56)

This table shows the distribution and characteristics of healthy subjects and individuals with stroke by age decades. Scores in the clinical scales are expressed in terms of mean and standard deviation (in parentheses)

A total of 355 healthy individuals (169 men and 186 women), with a mean age of 43.3 ± 18.6 years old were also enrolled (Table 1).

### Performance comparison

The motor performance of both groups evidenced significant differences in most of the spatiotemporal gait values. Healthy participants showed higher gait speed values in comparison with participants with stroke. Stride and step length were slightly higher in healthy individuals and stride and step time evidenced higher values in individuals post-stroke. Step width reflected higher values in individuals with stroke. Participants post-stroke also showed higher double support time in comparison to healthy individuals, which reached statistical significance in adults. Regarding kinematics values, participants with stroke evidenced higher trunk tilt compared with healthy controls. Other spatiotemporal and kinematic parameters evidenced different performance in both populations, which gave rise to multiple statistical significant differences (Tables 2 and 3).

**Table 2:** Mean values of gait parameters for healthy subjects (normative values)

	Age decade						
	10–19	20–29	30–39	40–49	50–59	60–69	≥70
Speed (m/s)	1.16 (0.15)	1.18 (0.15)	1.16* (0.12)	1.19** (0.15)	1.16** (0.19)	1.06** (0.17)	0.94** (0.19)
Stride length (m)	1.23** (0.11)	1.32 (0.11)	1.29** (0.10)	1.27** (0.14)	1.25** (0.14)	1.19** (0.16)	1.05** (0.16)
Stride time (s)	1.08** (0.09)	1.13 (0.10)	1.12 (0.10)	1.08** (0.09)	1.09** (0.12)	1.13** (0.10)	1.19 (0.12)
Step length (m)	0.63* (0.05)	0.67 (0.06)	0.65** (0.05)	0.64** (0.07)	0.62** (0.07)	0.60** (0.08)	0.52** (0.08)
Step time (s)	0.54** (0.05)	0.56 (0.05)	0.56 (0.05)	0.54** (0.05)	0.55** (0.06)	0.56** (0.05)	0.59 (0.07)
Step width (m)	0.11** (0.03)	0.12 (0.03)	0.12** (0.03)	0.11** (0.02)	0.12** (0.03)	0.10** (0.03)	0.13** (0.04)
Cadence (step/min)	111.87**(9.27)	107.43 (9.60)	107.87 (10.25)	112.37** (9.36)	111.15** (12.03)	107.56** (9.10)	102.04 (9.99)
Step asymmetry (m)	0.05** (0.08)	0.06 (0.13)	0.04* (0.02)	0.05* (0.03)	0.06* (0.13)	0.12 (0.26)	0.07 (0.08)
Swing time (s)	0.40 (0.05)	0.43 (0.14)	0.43* (0.15)	0.41 (0.12)	0.41 (0.14)	0.42 (0.12)	0.42** (0.07)
Double support time (s)	0.28 (0.07)	0.29 (0.08)	0.29* (0.07)	0.29** (0.09)	0.31** (0.10)	0.37 (0.19)	0.42 (0.19)
Trunk obliquity (°)	4.35 (1.36)	5.05 (1.66)	4.69* (1.18)	4.29 (1.25)	5.17 (2.82)	4.87** (1.52)	4.76** (1.31)
Trunk tilt (°)	6.29 (1.68)	6.26 (0.95)	6.35* (1.64)	5.90** (1.02)	5.28** (1.02)	5.47** (1.26)	5.55** (1.43)
Trunk rotation (°)	14.17** (3.95)	15.61 (3.95)	16.12* (3.65)	17.08* (3.82)	17.34 (3.11)	17.80 (5.25)	14.87** (3.89)
Pelvic obliquity (°)	8.35 (2.14)	6.46 (1.98)	7.63 (2.55)	7.49 (3.11)	7.72 (2.12)	7.41 (1.64)	8.22 (1.80)
Pelvic tilt (°)	17.34 (3.85)	13.68 (3.85)	16.08 (5.38)	16.75 (8.25)	17.08 (5.59)	19.13 (5.64)	19.67 (5.46)
Pelvic rotation (°)	11.12 (4.06)	10.16* (3.74)	11.04 (3.62)	13.08 (2.90)	10.15* (2.27)	11.72 (3.29)	11.03* (3.09)
Hip abduction-adduction (°)	10.19 (2.51)	10.09 (1.98)	9.39** (2.10)	11.26 (3.19)	11.95 (3.47)	11.56 (1.69)	12.58 (3.70)
Hip flexion-extension (°)	57.36 (6.33)	53.82 (6.06)	52.70 (5.76)	56.58 (4.76)	56.72* (5.72)	55.94* (7.71)	48.42 (8.65)
Hip height variation (cm)	0.06 (0.01)	0.07** (0.01)	0.07 (0.01)	0.06 (0.01)	0.06 (0.01)	0.06 (0.01)	0.05 (0.01)
Knee valgus-varus alignment (°)	18.42 (3.73)	19.52 (4.56)	17.65 (5.51)	19.17 (5.44)	21.68 (4.46)	21.61 (4.42)	22.81 (7.42)
Knee flexion-extension (°)	52.45 (4.19)	50.66 (7.66)	50.33** (4.85)	50.53 (5.58)	47.97 (5.80)	47.75 (5.27)	46.38 (6.24)
Knee height variation (cm)	0.16 (0.13)	0.10 (0.02)	0.10 (0.04)	0.10 (0.07)	0.09 (0.05)	0.08 (0.04)	0.11 (0.09)
Ankle height variation (cm)	0.17 (0.04)	0.20** (0.03)	0.20 (0.09)	0.21 (0.06)	0.17 (0.02)	0.17 (0.02)	0.17 (0.05)

Performance of healthy subjects divided by age decade in all spatiotemporal and kinematic gait parameters.

All measures are expressed in terms of mean and standard deviation (in parentheses). *: *p* < 0.05. **: *p* < 0.01

**Table 3:** Mean values of gait parameters for individuals post-stroke

	Age decade						
	10–19	20–29	30–39	40–49	50–59	60–69	≥70
Speed (m/s)	1.13 (0.22)	1.18 (0.34)	1.03* (0.26)	0.90** (0.31)	0.81** (0.28)	0.75** (0.37)	0.67** (0.14)
Stride length (m)	1.40** (0.11)	1.30 (0.26)	1.16** (0.20)	1.07** (0.25)	1.00** (0.28)	0.92** (0.37)	0.82** (0.18)
Stride time (s)	1.31** (0.34)	1.15 (0.19)	1.17 (0.19)	1.26** (0.28)	1.31** (0.27)	1.35** (0.30)	1.22 (0.11)
Step length (m)	0.68* (0.08)	0.64 (0.10)	0.58** (0.10)	0.55** (0.12)	0.49** (0.14)	0.46** (0.19)	0.41** (0.08)
Step time (s)	0.64** (0.18)	0.58 (0.11)	0.59 (0.10)	0.63** (0.14)	0.65** (0.12)	0.68** (0.15)	0.61 (0.06)
Step width (m)	0.15** (0.05)	0.13 (0.03)	0.17** (0.06)	0.18** (0.05)	0.21** (0.07)	0.18** (0.05)	0.17** (0.06)
Cadence (step/min)	97.66** (15.79)	107.41 (15.90)	105.02 (15.07)	99.22** (20.24)	94.34** (14.48)	92.23** (17.62)	98.96 (9.79)
Step asymmetry (m)	0.28** (0.24)	0.04 (0.04)	0.05* (0.04)	0.08* (0.08)	0.07* (0.05)	0.06 (0.05)	0.10 (0.05)
Swing time (s)	0.33 (0.13)	0.47 (0.09)	0.38* (0.08)	0.41 (0.07)	0.36 (0.11)	0.41 (0.06)	0.33** (0.13)
Double support time (s)	0.32 (0.07)	0.26 (0.19)	0.37* (0.20)	0.46** (0.21)	0.47** (0.17)	0.46 (0.23)	0.45 (0.15)
Trunk obliquity (°)	5.85 (2.47)	4.97 (1.74)	6.19* (2.51)	6.44 (3.72)	6.97 (3.64)	8.27** (3.61)	7.58** (2.54)
Trunk tilt (°)	8.04 (1.70)	7.95* (1.99)	8.32* (2.72)	9.15** (3.10)	7.96** (2.92)	9.99** (5.47)	9.49** (3.79)
Trunk rotation (°)	21.7** (4.45)	16.82 (4.29)	22.42* (9.23)	21.93* (6.95)	22.24 (8.81)	17.06 (5.56)	20.25 (3.04)
Pelvic obliquity (°)	9.27 (1.64)	6.88 (2.60)	9.55 (3.07)	9.52 (2.85)	9.16 (2.17)	7.85 (1.64)	9.22 (4.05)
Pelvic tilt (°)	19.86 (4.53)	13.84 (5.51)	23.29* (9.87)	20.74 (7.74)	23.74 (9.77)	20.20 (8.68)	21.56 (8.67)
Pelvic rotation (°)	15.45 (4.75)	15.20* (7.09)	14.68 (6.33)	16.01 (5.94)	15.66* (7.06)	12.12 (5.45)	13.63* (1.81)
Hip abduction-adduction (°)	11.74 (3.22)	10.58 (2.59)	13.33** (2.45)	13.00 (3.52)	12.05 (2.09)	12.02 (2.89)	14.20 (2.06)
Hip flexion-extension (°)	59.49 (9.03)	56.49 (6.80)	55.22 (7.82)	52.84 (7.22)	49.28* (9.49)	49.52* (8.18)	45.30 (9.86)
Hip height variation (cm)	0.20 (0.24)	0.37** (0.32)	0.07 (0.02)	0.18 (0.34)	0.06 (0.02)	0.07 (0.06)	0.05 (0.01)
Knee valgus-varus alignment (°)	22.17 (11.84)	19.41 (5.98)	21.79 (7.10)	19.36 (6.11)	18.61 (4.40)	19.95 (6.01)	22.97 (4.97)
Knee flexion-extension (°)	49.37 (7.36)	45.03 (16.58)	43.74** (8.14)	50.06 (12.75)	44.89 (8.83)	39.60 (15.53)	41.40 (8.89)
Knee height variation (cm)	0.19 (0.16)	0.09 (0.03)	0.15 (0.16)	0.10 (0.03)	0.13 (0.12)	0.18 (0.24)	0.12 (0.05)
Ankle height variation (cm)	0.23 (0.11)	0.33** (0.13)	0.19 (0.05)	0.23 (0.13)	0.17 (0.05)	0.17 (0.07)	0.14 (0.05)

Performance of individuals with stroke divided by age decade in all spatiotemporal and kinematic gait parameters.

All measures are expressed in terms of mean and standard deviation (in parentheses).*: *p* < 0.05. **: *p* < 0.01

The effects of age were also evidenced in the performance of both groups. All the participants showed almost invariable gait speed, which decreased for those older than 60. This negative effect of age on gait speed was evidenced in both healthy (*r* = − 0.377; *p* = 0.000) and participants with stroke (*r* = − 0.471; *p* = 0.000) (Tables 2 and 3). Stride and step length showed a clear decrease with age in healthy adults (*r* = − 0.385; *p* = 0.000 and *r* = − 0.423; *p* = 0.000, respectively) and adults with stroke (*r* = − 0.519; *p* = 0.000 and *r* = − 0.519; *p* = 0.000, respectively). In contrast, stride and step time remained more stable for all the participants. Healthy participants showed increased double support time at older ages (*r* = 0.340; *p* = 0.000), which was also reflected by participants post-stroke (*r* = 0.295; *p* = 0.009). Knee flexion-extension also evidenced a decrease with age in the healthy group (*r* = 0.334; *p* = 0.001), which was not reflected by individuals with stroke (*r* = − 0.150; *p* = 0.187). A similar age-effect was detected for trunk tilt. While healthy individuals showed decreasing trunk tilt with age (*r* = − 0.232; *p* = 0.025), individuals post-stroke did not showed a clear tendency (*r* = 0.162; *p* = 0.152). Other spatiotemporal and kinematic parameters did not show a clear age-related tendency.

### Concurrent validity

Significant correlations were detected between all the clinical tests and all spatiotemporal parameters obtained by the Kinect v2-based gait analysis, but for the step asymmetry (Table 4). Strong correlations were found between all the clinical tests and speed, stride, step, and cadence, but those between the DGI and stride and step length, which were very strong, and those between the 10MWT and stride time and cadence, which were moderate. Correlations between clinical scales and double support time were moderate, while correlations between clinical scales and step width and swing time were weak, but for that between the 10MWT and the swing time, which was moderate.

**Table 4:** Concurrent validity of the spatiotemporal and kinematic parameters with clinical measures

	Dynamic gait index	1-min walking test	10-m walking test
Speed (m/s)	*r* = 0.793**	*r* = 0.754**	*r* = −0.675**
Stride length (m)	*r* = 0.822**	*r* = 0.684**	*r* = −0.726**
Stride time (s)	*r* = − 0.645**	*r* = − 0.608**	*r* = 0.596**
Step length (m)	*r* = 0.806**	*r* = 0.707**	*r* = − 0.721**
Step time (s)	*r* = −0.658**	*r* = − 0.622**	*r* = 0.602**
Step width (m)	*r* = −0.400**	*r* = −0.342**	*r* = 0.332**
Cadence (step/min)	*r* = 0.614**	*r* = 0.654**	*r* = −0.557**
Step asymmetry (m)	*r* = −0.092	*r* = −0.028	*r* = 0.053
Swing time (s)	*r* = 0.376**	*r* = 0.274*	*r* = −0.475**
Double support time (s)	*r* = −0.554**	*r* = −0.551**	*r* = 0.505**
Trunk obliquity (°)	*r* = −0.539**	*r* = −0.520**	*r* = 0.571**
Trunk tilt (°)	*r* = −0.599**	*r* = −0.427**	*r* = 0.688**
Trunk rotation (°)	*r* = 0.136	*r* = −0.088	*r* = 0.014
Pelvic obliquity (°)	*r* = −0.180	*r* = −0.147	*r* = 0.073
Pelvic tilt (°)	*r* = −0.344**	*r* = −0.297*	*r* = 0.317**
Pelvic rotation (°)	*r* = −0.056	*r* = 0.021	*r* = 0.049
Hip abduction-adduction (°)	*r* = −0.342**	*r* = −0.184	*r* = 0.281*
Hip flexion-extension (°)	*r* = 0.436**	*r* = 0.437**	*r* = −0.241*
Hip height variation (cm)	*r* = 0.191	*r* = 0.368**	*r* = −0.173
Knee valgus-varus alignment (°)	*r* = 0.034	*r* = 0.080	*r* = −0.037
Knee flexion-extension (°)	*r* = 0.255*	*r* = 0.135	*r* = −0.296**
Knee height variation (cm)	*r* = 0.013	*r* = −0.036	*r* = −0.126
Ankle height variation (cm)	*r* = 0.436**	*r* = 0.536**	*r* = −0.342**

Significant correlations of variable strength emerged between spatiotemporal and kinematic parameters obtained by the Kinect v2-based gait analysis and standardized clinical scales. *: *p* < 0.05. **: *p* < 0.01

Significant correlations were also found between the clinical scales and the kinematic parameters. Strength of the correlation with clinical scales was moderate for hip flexion-extension, ankle height variation and trunk obliquity and tilt, with the exception of that between trunk tilt and the 10MWT, which was strong and between hip flexion-extension and ankle height variation with 10MWT, which were weak. Correlations between pelvic tilt, hip abduction, and knee flexion-extension with clinical scales were weak but those between hip abduction and knee flexion-extension with the 1mWT, where no significant correlations were found. An addition moderate correlation was also found between hip height variation and the 1mWT. No correlations were found for trunk rotation, pelvic obliquity and rotation, knee valgus-varus alignment and knee height variation.

### Inter and intra-rater reliability, standard error of measurement, and minimal detectable change

Results evidenced excellent inter and intra-rater reliability for all measures but for step asymmetry, inter-rater reliability of hip abduction, and intra-rater reliability of knee height variation, which were all good (Table 5).

**Table 5:** Inter and intra-rater reliability, standard error of measurement, and minimal detectable change of gait parameters

	Inter-rater reliability	Intra-rater reliability	Standard error of measurement	Minimal detectable change
Speed (m/s)	*r* = 0.970**	*r* = 0.970**	0.04	0.12 (12.4%)
Stride length (m)	*r* = 0.975**	*r* = 0.982**	0.03	0.09 (8.1%)
Stride time (s)	*r* = 0.948**	*r* = 0.958**	0.03	0.10 (8.1%)
Step length (m)	*r* = 0.977**	*r* = 0.970**	0.02	0.06 (10.6%)
Step time (s)	*r* = 0.947**	*r* = 0.960**	0.02	0.05 (7.8%)
Step width (m)	*r* = 0.943**	*r* = 0.894**	0.01	0.03 (23.9%)
Cadence (step/min)	*r* = 0.939**	*r* = 0.951**	3.17	8.79 (8.6%)
Step asymmetry (m)	*r* = 0.636*	*r* = 0.767**	0.03	0.09 (103.4%)
Swing time (s)	*r* = 0.834**	*r* = 0.802**	0.04	0.11 (33.0%)
Double support time (s)	*r* = 0.904**	*r* = 0.814**	0.08	0.22 (50.7%)
Trunk obliquity (°)	*r* = 0.846**	*r* = 0.805**	1.01	2.80 (35.7%)
Trunk tilt (°)	*r* = 0.905**	*r* = 0.886**	0.77	2.15 (25.1%)
Trunk rotation (°)	*r* = 0.913**	*r* = 0.885**	2.62	7.25 (28.1%)
Pelvic obliquity (°)	*r* = 0.821**	*r* = 0.924**	2.10	5.81 (54.1%)
Pelvic tilt (°)	*r* = 0.877**	*r* = 0.965**	6.07	16.82 (78.4%)
Pelvic rotation (°)	*r* = 0.968**	*r* = 0.942**	2.42	6.70 (35.8%)
Hip abduction-adduction (°)	*r* = 0.787**	*r* = 0.838**	2.05	5.69 (42.0%)
Hip flexion-extension (°)	*r* = 0.889**	*r* = 0.862**	3.26	9.04 (16.5%)
Hip height variation (cm)	*r* = 0.746**	*r* = 0.856**	0.01	0.04 (56.9%)
Knee valgus-varus alignment (°)	*r* = 0.881**	*r* = 0.915**	2.48	6.90 (32.5%)
Knee flexion-extension (°)	*r* = 0.893**	*r* = 0.938**	2.59	7.17 (15.0%)
Knee height variation (cm)	*r* = 0.829**	*r* = 0.711**	0.08	0.22 (110.7%)
Ankle height variation (cm)	*r* = 0.922**	*r* = 0.859**	0.02	0.05 (29.8%)

Inter and intra-rater reliability of the spatiotemporal and kinematic parameters. Minimal detectable change is expressed in absolute values and percentage (in parentheses). *: *p* < 0.05. **: *p* < 0.01

The minimal detectable change for spatiotemporal measures was excellent for step time and stride measures, moderate for speed, step length, and poor for step asymmetry, swing, and double support time. For kinematic measures, minimal detectable change was moderate for trunk tilt and rotation, hip and knee flexion, and ankle height variation, and was poor for the all other measures.

### Ability to discriminate between fallers and non-fallers

Eighteen participants were categorized as more likely to fall and the remaining were categorized with lower fall risk. Significant differences were found in all spatiotemporal parameters, but in step asymmetry and several kinematic parameters, as trunk obliquity and tilt, pelvic tilt, hip abduction and flexion, and ankle height variation (Table 6). Results indicated that participants with higher fall risk had remarkably lower speed and cadence, shorter stride and step length and time, and higher double support time. Step asymmetry was, in contrast, similar in both groups. Fall risk was also associated to increased trunk and pelvic obliquity and tilt, and to decreased hip flexion-extension and ankle height variation. Other kinematic measures were similar between groups and did not reach statistical significance. Finally, results of the ROC analysis showed that speed, stride and step length, stride and step time and cadence had high sensitivity and specificity and, consequently, were good predictors of fall risk. In turn, swing and double support time, trunk obliquity and tilt, hip flexion, and ankle height variation proved to be fair predictors of fall risk.

**Table 6:** Ability of gait parameters to discriminate between fallers and non-fallers

	Higher fall risk	Lower fall risk	Statistical significance	ROC
Speed (m/s)	0.55 (0.32)	1.00 (0.23)	*p* = 0.000	0.866
Stride length (m)	0.76 (0.37)	1.14 (0.20)	*p* = 0.000	0.826
Stride time (s)	1.52 (0.32)	1.17 (0.15)	*p* = 0.000	0.857
Step length (m)	0.38 (0.18)	0.57 (0.10)	*p* = 0.000	0.833
Step time (s)	0.75 (0.15)	0.58 (0.07)	*p* = 0.000	0.846
Step width (m)	0.21 (0.07)	0.17 (0.05)	*p* = 0.015	0.668
Cadence (step/min)	81.88 (15.71)	103.92 (12.51)	*p* = 0.000	0.858
Step asymmetry (m)	0.09 (0.05)	0.07 (0.10)	*p* = 0.401	0.685
Swing time (s)	0.31 (0.14)	0.40 (0.08)	*p* = 0.003	0.715
Double support time (s)	0.56 (0.18)	0.37 (0.17)	*p* = 0.000	0.797
Trunk obliquity (°)	9.54 (4.72)	5.90 (2.14)	*p* = 0.000	0.759
Trunk tilt (°)	11.53 (5.02)	7.75 (2.14)	*p* = 0.000	0.762
Trunk rotation (°)	22.50 (7.89)	20.09 (7.13)	*p* = 0.228	0.612
Pelvic obliquity (°)	9.83 (3.40)	8.50 (2.15)	*p* = 0.052	0.590
Pelvic tilt (°)	25.24 (12.35)	20.03 (7.15)	*p* = 0.029	0.606
Pelvic rotation (°)	15.67 (6.81)	14.67 (5.95)	*p* = 0.552	0.541
Hip abduction-adduction (°)	13.43 (3.43)	11.95 (2.31)	*p* = 0.039	0.612
Hip flexion-extension (°)	45.97 (10.91)	53.54 (7.79)	*p* = 0.002	0.725
Hip height variation (cm)	0.06 (0.03)	0.13 (0.22)	*p* = 0.189	0.651
Knee valgus-varus alignment (°)	18.75 (6.69)	20.05 (5.13)	*p* = 0.390	0.577
Knee flexion-extension (°)	44.35 (8.07)	46.12 (11.36)	*p* = 0.552	0.549
Knee height variation (cm)	0.13 (0.10)	0.13 (0.14)	*p* = 0.972	0.575
Ankle height variation (cm)	0.14 (0.04)	0.21 (0.10)	*p* = 0.004	0.760

Significant correlations of variable strength emerged between spatiotemporal and kinematic parameters obtained by the Kinect v2-based gait analysis and standardized clinical scales. ROC: receiver operating characteristic

## Discussion

This study investigates the sensitivity of an open-access Kinect v2-based gait analysis system to motor disability and risk of fall, its concurrent validity with clinical scales, inter and intra-rater reliability, minimal detectable change. Results revealed different performance not only between healthy subjects and individuals with stroke, but also between individuals post-stroke with and without risk of fall and evidenced significant but variable concurrent validity of gait parameters with clinical scales, good to excellent inter and intra-rater reliability, and also variable minimal detectable change. These results support the validity and reliability of the Kinect v2-based gait analysis, which could complement clinical gait evaluations.

Mean speed values exhibited by healthy controls in our study were lower than those reported in other studies [47–49]. This could be derived from an effect of the acceleration and deceleration phases [50], which are conventionally excluded from analysis to investigate gait at a constant speed [50]. In our study, although the effect of the acceleration phase could be partially avoided, as the first meter of walking was not registered by the Kinect v2, the deceleration phase was almost entirely included for analysis, given that participants reduced speed as they approached the device. This difference in the procedure, derived from the technical limitations of the Kinect v2, might have affected the results and, therefore, could explain the incongruences with previously reported normative values [49, 51]. As a proof, comparable results to ours have been shown with comparable procedures [50].

However, in spite this discrepancy and the heterogeneity in the distribution of participants by age decade (more pronounced in the stroke group), the well-known gait decline with age [52] was evidenced by both healthy controls and participants post-stroke. In addition, differences between the performances of both populations, which are largely supported by the literature, were also evident. The slower speed and cadence and shorter stride length shown by participants with stroke are characteristic of hemiparetic gait post-stroke [53]. The increased trunk and pelvic kinematics detected in this group could be explained by an impaired coordination and decreased muscle strength, which are common after stroke [54]. The limited hip and knee flexion-extension detected in participants with stroke is also a distinctive impairment in this population [55]. The inclusion of both sides for analysis could have prevented even greater differences between groups, as an increased flexion of the less affected leg of individuals with stroke could have overcompensated the limited flexion in the more affected side, as previously reported [53]. Nonetheless, the results of participants post-stroke should be examined with caution as they have been reported to show high variability [56] and, in addition, a wide deviation is expected in hemiplegic individuals [2]. Even so, the performance of participants with stroke was similar to that reported by other studies including similar population [57–59].

The concurrent validity of the Kinect v2-based gait analysis with traditional scales is comparable not only to that of the clinical tools used in the study [60] but also to laboratory-grade and gold-standard systems [61, 62]. The sign of the correlations, in turn, supported the consistency of the gait measures, as better performance in all gait parameters were related to better performance in the clinical scales. For instance, higher gait speed was associated to higher score in the DGI, higher distance walked in the 1mWT, and shorter time on the 10MWT. The inter-rater and intra-rater reliability of the gait measures was comparable to those obtained by both clinical tools, such as the 10MWT [63, 64] or the DGI [38], and instrumented systems [25, 26] in a stroke sample, and more importantly, are supported by preliminary studies exploring spatiotemporal [25] and kinematic gait analysis [26] with the Kinect v2. Accuracy of spatiotemporal analysis, as represented by the standard error of measurement, is in line with previous reports [31]. Although results might vary according to the population being tested, the sensitivity to detect small changes of the Kinect v2-based gait speed measurement was comparable not only to walking tests including similar distances, such as the three-meter walking test [65] and the four-meter gait speed [66], but also to instrumented walkways [65] and optical motion tracking systems [67]. Sensitivity to changes in step and stride measures was also comparable to optical motion tracking [67] and poorer for other parameters. The minimal detectable change of some gait parameters should be highlighted as they allow for detecting changes that could be clinically important. For instance, the minimal detectable change of the Kinect v2-based system in gait speed was 0.12 m/s, which is almost a third of that using a stopwatch [68]. Importance of this accuracy relies on its potential capacity to detect minimal clinically important changes in the gait speed, which have been established by different studies as being 0.13 m/s [69], 0.16 m/s [70], and 0.19 m/s [71] in individuals with subacute stroke and different disabilities.

The ability of almost all Kinect v2-based gait parameters to examine fall risk should be highlighted as increased fall risk is a well-known affliction after stroke during rehabilitation [72] and after discharge [73]. Other systems based on wearable inertial sensors [74] and Kinect-based data have been presented to assess fall risk [75]. The Kinect v2-based gait analysis could successfully identify those individuals who might need more rehabilitative attention to prevent future falls according to the BBS in a shorter time than not only the mentioned instrumented systems but also than the clinical scale itself.

All these results support that, despite the limited sensitivity of the kinematic parameters, the Kinect v2-based gait analysis was able to successfully evidence the performance of both healthy controls and individuals post-stroke, showing good concurrent validity with clinical scales, excellent reliability, and excellent ability to discriminate fall risk after stroke. These features, together with the low-cost, availability, and portability of the device, could support the use of the Kinect v2-based gait analysis as a low-cost alternative to laboratory-grade systems in the clinical setting, to complement gait assessment.

## Conclusions

The Kinect v2-based gait analysis successfully characterized a cohort of individuals with stroke in comparison to age-matched healthy subjects, showed concurrent validity with clinical scales, excellent reliability, variable sensitivity, and also excellent ability to identify risk fall, which supports its use as a low-cost alternative to laboratory-grade systems under certain circumstances to complement clinical gait assessment.

## Data Availability

The datasets used and/or analyzed during the current study are available from the corresponding author on reasonable request.

## Abbreviations

10MWT: 10-m Walking Test
1mWT: 1-min Walking Test
BBS: Berg Balance Scale
DGI: Dynamic Gait Index
